# Health Benefits of a Standardized *Ginkgo biloba* Extract Associated With Phosphatidylserine in Alleviating Mental Stress and Cognitive Performance: Results From Two Exploratory Randomized Trials

**DOI:** 10.1002/fsn3.72001

**Published:** 2026-06-11

**Authors:** Kenny Hawkins, Justine Davis, Cristal Abreu Matias, Gabriel Wilson, Ryan Lowery, Paola Misiano, Giovanna Petrangolini, Jacob Wilson

**Affiliations:** ^1^ Applied Science & Performance Institute Tampa Florida USA; ^2^ Department of Pharmacological and Biomolecular Sciences Università Degli Studi di Milano Milan Italy; ^3^ Medical Department Indena SpA Milan Italy

**Keywords:** cognition, *Ginkgo biloba* extract, neuroprotective activities, phosphatidylserine, Virtiva Plus

## Abstract

Mental health is threatened every day by the stressors of life: frenetic work rhythms, stressful conditions can generate anxiety, depression, cognition, and sleep problems. Among natural products with properties to support mental health, *Gingko biloba* extract with phosphatidylserine (GBP) was exploited with the aim of evaluating its benefits in cognitive function, mood regulation, and overall quality of life. *Gingko Biloba* has been widely used to improve mental functions, while phosphatidylserine displayed neurotransmitter modulation in several studies. Two exploratory, registered RCT clinical studies were performed. In the first 480 mg/day of GBP, or placebo, was supplemented to healthy volunteers for 4 weeks. Mood states and cognitive performance were evaluated at 60 and 180 min (single dose), and after 4 weeks of supplementation. In the second trial adults experiencing moderate stress received GBP240 (240 mg/day) or GBP480 (480 mg/day) for 4 weeks, and perceived stress, anxiety, mood states, and cognitive performance were evaluated. Following both acute (single dose) and chronic (4‐week) GBP480 supplementation, participants exhibited improvements in delayed verbal memory, and reaction time versus placebo, suggesting a possible beneficial impact on cognitive functions. The second study confirmed the GBP benefits on mood states and a visually consistent downward trend in reaction times across all four cognitive performance tests at both GBP dosages, suggesting improved processing speed in response to various stimuli. On the basis of the results obtained in the first study, the results on cognitive models on the second study were more modest than expected. However, the results described in the present research confirmed previous reported advantages of the unique association of *Gingko Biloba* and phosphatidylserine, so combining antioxidant, neuroprotective, and nootropic properties of both ingredients. Further research is needed to consolidate this preliminary evidence.

## Graphical abstract

Promising results by the supplementation of *Gingko biloba* extract associated with Phosphatidylserine in two exploratory randomized trials on brain health and well‐being, so combining antioxidant, neuroprotective, and nootropic properties of both ingredients.

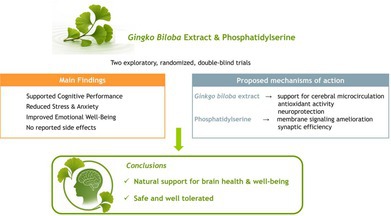

## 1 Introduction

Maintaining cognitive and emotional well‐being across the lifespan is fundamental to promoting overall quality of life. Polyphenolic compounds were extensively studied for their antioxidant and anti‐inflammatory properties, contributing to a well‐being status; however, recent literature highlighted the risks of an excessive intake of polyphenols that may not be so beneficial (Özdemir and Demir 2025; Kasapoğlu et al. 2026). Preserving optimal brain health reduces the risk of cognitive decline, supports mental resilience, and enhances productivity and creativity. However, a wide array of factors, such as psychological stress, personal and societal anxieties, and insufficient sleep, can impair mental health. Environmental pollutants, such as heavy metals and pesticides, can represent a danger for several organs, like the liver, kidneys, bones, and also the brain (Hoff et al. 2020; Caglayan et al. 2019). All those factors may contribute to symptoms such as memory decline, reduced concentration, poor impulse control, and an impaired ability to cope with challenges.

Mood and anxiety disorders in adulthood, including depression, are linked to numerous adverse outcomes, such as a heightened risk of recurring mental health conditions, substance use, reduced educational attainment, unemployment, and an increased likelihood of suicidal thoughts and behaviors (Duffy et al. 2019).

Given these challenges, there is growing interest in natural strategies to support mental and cognitive health. Recent advanced techniques of integrated LC–MS/MS, GC–MS, enzyme inhibition, molecular docking, and bioinformatics allowed the discovery of potential properties of plants and flowers (Lotharius et al. 2014; Zor et al. 2025). Among these, dietary supplements have emerged as promising interventions due to their capacity to influence neurotransmitter pathways, support mood regulation, and improve cognitive performance (Muscaritoli 2021). Among the most popular botanical options is *Gingko biloba* (GB), an extract from the leaves of the secular ginkgo tree known for anti‐anxiolytic and cognitive boosting properties (Brondino et al. 2013). Despite these promising effects, the clinical application of GB has been limited by its poor solubility and bioavailability (Akanchise and Angelova 2023).

Emerging clinical and mechanistic evidence supports the efficacy of GB extract in enhancing memory, executive function, and mood in both healthy individuals and those with mild cognitive or mood disorders (Achete de Souza et al. 2020; Barbalho et al. 2022; DeFeudis 1991; Field and Vadnal 1998; Franke et al. 2014; Li et al. 2023; Pagotto et al. 2024; Silberstein et al. 2011; Singh et al. 2019; Tomino et al. 2021; Unger 2013; Zhang et al. 2016). The leaves of GB are among the most widely utilized sources for supplements, which are widely used for their potential health benefits in supporting memory, cognition, and managing conditions such as Alzheimer's disease (AD), Parkinson's disease (PD), and dementia (Barbalho et al. 2022; Pagotto et al. 2024; Ma et al. 2022). Similar to bilberry (Kara et al. 2025), beneficial effects on vascular endothelium were demonstrated for GB, which showed inhibitory activity on expression of inducible nitric oxide synthase (iNOS) (Cheung et al. 1999), producing microvascular antioxidant protection. Inhibition of endothelial expression of intercellular adhesion molecule 1 (ICAM‐1) and increase in telomerase activity (Dong et al. 2007) were also displayed by GB. However, the variability in outcomes across studies underscores the need for further investigation, particularly with standardized formulations.

The potential of ginkgo extract can be further enhanced by a rational combination with phosphatidylserine, a phospholipid used in dietary supplementation for its nootropic effects (Singh et al. 2019). Phosphatidylserine (PS) is the main acidic phospholipid found in the brain and is located primarily in the inner layer of the plasma membrane. Oral intake of PS can influence the structure of neuronal membranes, cellular metabolism, and various neurotransmitter systems. Several clinical studies have shown that PS extracted from bovine cortex can provide significant benefits to brain functions, particularly those that tend to decline with age, such as memory, learning, vocabulary skills, and concentration. Moreover, PS appears to help regulate neuronal excitability and neurotransmitter activity, processes that are essential for cognitive functions like learning and memory, and it is orally supplemented for these beneficial effects (Ma et al. 2022; Glade and Smith 2015; Kim et al. 2014). This botanical association that comes from the “smart synergistic combination” of a highly standardized *Ginkgo biloba* extract and phosphatidylserine (GBP, Virtiva Plus) resulted in the following bioactive components: ginkgoflavonglucosides, ginkgoterpenes, and phosphatidylserine, with beneficial effects on cognitive health and emotional balance. Thus, other than the GB properties described above, GB active compounds are known to have a strong antioxidant effect capable of conferring protection to the cardiovascular system, but also to brain function by improving cerebral circulation (Lotharius et al. 2014).

A previous clinical study had already demonstrated its effectiveness in optimizing memory speed and quality after a single administration of GBP at a dosage of 480 mg in healthy volunteers (Kennedy et al. 2007). This was more significant than ginkgo extract alone or its combination with other phospholipids (e.g., phosphatidylcholine). Moreover, in a randomized, double‐blind, placebo‐controlled human study in professional young volleyball athletes, GBP supplementation was able to modulate the increase of cortisol (the so‐called “stress hormone”) associated to sport training, suggesting a beneficial effect on stress and brain performances during stressful activity requiring mental alertness, including sport training and competition (Di Pierro et al. 2016). The present research aimed to clinically evaluate the efficacy of GBP supplementation, both in different dosages and compared to placebo, in improving emotional balance and cognitive performance. Outcomes of interest included concentration, alertness, reaction time, and both verbal and visual memory.

## 2 Materials and Methods

### 2.1 Supplement

The GBP supplement (GBP as Virtiva Plus, supplied by Indena S.p.A., Milan, Italy) used in the study is an association between *Ginkgo biloba* L. standardized extract (about 25%) and lecithin (sunflower origin: about 75%), containing 20% phosphatidylserine. The association is standardized to contain: ≥ 5% ginkgoflavonglycosides, ≥ 0.5% ginkgoterpenes, and ≥ 12% phosphatidylserine, evaluated by HPLC.

For the first RCT study, GBP 240 mg and Placebo capsules were manufactured by Nature's Value (Coram, New York, USA). Each GBP capsule contained 120 mg of the active ingredient, rice flour, and magnesium stearate as inactive ingredients. Placebo capsules contained the same inactive ingredients as GBP capsules. GBP and Placebo capsules were manufactured by dry mixing the respective components and filling the mix into size 1 hydroxypropyl methylcellulose vegetarian hard capsules. To maintain blinding integrity, ensuring that participants and investigators remained unaware of group assignments, green colored hydroxypropyl methylcellulose vegetarian hard capsules were used for both GBP and Placebo. GPB and Placebo capsules were tested for appearance, active substance HPLC identification, active substance HPLC assay (only for GPB capsules), heavy metals (lead, cadmium, arsenic, and mercury), and microbiological quality. Ginkgoflavonglucosides were used as markers of the active ingredient for HPLC identification and assay.

For the second RCT study, GBP 120 and 240 mg capsules were manufactured by Liquid Capsule Manufacturing LCC (Tampa, Florida, USA).

The same manufacturing process and the same capsule components were applied for the second study. As for the first study, capsules were tested for appearance, active substance HPLC identification and assay, heavy metals (lead, cadmium, arsenic, and mercury), and microbiological quality.

### 2.2 Clinical Studies

The human benefits were evaluated through two exploratory randomized clinical studies, as described.

Both studies were performed by the Applied Science and Performance Institute (ASPI Labs) (ClinicalTrials.gov ID: First Study NCT06309914; Second Study NCT06583941), in agreement with the protocols approved by an external Institutional Review Board (First Study: Advarra IRB, ID: Pro00074459, September 2023; Second Study: Advarra IRB, ID Pro00081043, August 2024), and in compliance with the Helsinki Declaration, International Conference on Harmonization Good Clinical Practice guidelines (ICH E6‐R2) and applicable local regulatory requirements and laws.

#### 2.2.1 First Study: RCT Versus Placebo

A randomized, double‐blind, placebo‐controlled pilot study (NCT06309914) was performed. The trial was part of a broader investigation involving four arms: GBP, grape seed extract, bilberry extract, and placebo (Kara et al. 2025). Only data from the GBP and placebo arms are presented here. The aim of this pilot study was to evaluate the effects of 480 mg daily of GBP (taken as 240 mg twice per day; hereafter, referred to as GBP480) on cognitive performance, mood state, and the occurrence of adverse events in response to acute (60–180 min) and daily 4‐weeks of supplementation. Healthy men and women, 25–55 years of age, were enrolled. Subjects were randomly divided into two study groups: Placebo or GBP480.

Healthy adult participants aged 25 to 55 were recruited across the United States via remote screening. Eligibility criteria included the ability to read and sign informed consent, access to a Wi‐Fi–enabled device, and willingness to comply with study procedures. Exclusion criteria included developmental disabilities, cognitive impairment, pregnancy, significant cardiovascular, neurological, gastrointestinal, metabolic, or oncological conditions, alcohol or drug misuse, and the use of medications affecting digestion.

After prescreening, subjects were randomized into two groups: one received GBP480, the other received the placebo. The first study visit was conducted under fasting conditions. Participants ingested their assigned intervention (two capsules totaling 480 mg GBP or placebo), and outcome assessments were conducted at baseline, 60 min, and 180 min postdose to evaluate acute effects. Participants then continued daily supplementation for 4 weeks, after which final assessments were performed in a fasted state.

Primary and secondary objectives were:

- *Mood*: Abbreviated Profile of Mood States (POMS), that is, a 40‐item questionnaire version where participants rate each item on a 5‐point Likert scale with anchors ranging between “Not at all” to “Extremely.” Items are combined to form seven separate subscales. The subscale scores are then combined to form an overall measure of affect that is labeled as total mood disturbance (TMD). A lower score indicates lower mood disturbance, while a higher score indicates increased mood disturbance (Grove and Prapavessis 1992).
- *Cognitive Performance*: Evaluated using the CNS Vital Signs computerized testing platform (Morrisville, North Carolina, USA) (CNS Vital Signs 2025), which assessed multiple domains including visual and verbal memory, motor speed (via finger tapping), processing speed (symbol digit coding), executive function (shifting attention, Stroop test), and sustained attention (4‐part and continuous performance tests).
- *Safety*: Adverse events and tolerability were assessed biweekly throughout the 4‐week study period.

#### 2.2.2 Second Study: RCT Versus 2 Doses

An exploratory randomized, double‐blind, parallel‐group clinical trial (NCT06583941) was designed to assess the dose‐dependent effects of GBP240 (240 mg/day) and GBP480 (480 mg/day) of GBP on perceived stress, anxiety, mood states, and cognitive performance in adults experiencing moderate stress.

Inclusion criteria were individuals (males and females) moderately stressed aged 50 to 70 years (both limits inclusive), with body mass index (BMI) value of 18.5–29.99 kg/m^2^, willing and able to understand and sign the informed consent document and to comply with study protocol (scheduled visits, supplementation, and study requirements); not consuming cognitive enhancement supplements 7 days prior to the end of the study. Moderate stress was defined as a Perceived Stress Scale (PSS) score ≥ 13 to < 27.

Exclusion criteria were subjects presenting developmental disability or cognitive impairment, history of cardiological, neurological, or gastrointestinal/metabolic disorders, malignancy, illnesses, pregnancy, alcohol or drug abuse, and use of medications that impact digestion.

Briefly, participants were randomly assigned to one of the two blinded groups: GBP240 (120 mg per capsule/240 mg per day) and GBP480 (240 mg per capsule/480 mg per day), two daily capsules for 4 weeks. The dosage was divided into two equal servings, taken twice daily. Baseline Tests for cognitive performance, stress, and mood states were taken prior to commencement of supplementation. After 4 weeks of supplementation, tests were repeated. At the 4‐week timepoint, participants consumed a single serving of the study treatment with breakfast and completed testing 3 h later. Up to 30 individuals were enrolled, of whom 28 completed the study. The average study period for each subject was approximately 5 weeks.

The Primary Objective was to evaluate the dose–response efficacy of GBP (480 vs. 240 mg) on subjective metrics of stress and mood. The Secondary Objectives included assessing cognitive performance through computerized tasks measuring memory, attention, processing speed, executive function, and psychomotor abilities. A battery of cognitive tests was used for these evaluations, including both visual and verbal memory, finger tapping, symbol‐digit coding, Stroop test, shifting attention, and continuous performance tasks. These cognitive domains were selected based on CNS Vital Signs validation on computerized assessments on stressed and aging populations, with memory and processing speed prioritized as primary outcomes most sensitive to intervention, while executive function and attention measures were included as exploratory secondary assessments. Moreover, the observation and recording of adverse events (AE) experienced by study subjects after consuming the first dose of the study treatment and continuing through the final day of treatment consumption.

All participants completed the following validated surveys as outcome measures to assess stress, emotional state, mood, cognitive function, and overall well‐being:

- Perceived Stress Scale—10 (PSS‐10). The Perceived Stress Scale (PSS‐10) is a 10‐item questionnaire assessing the degree to which participants perceive situations in their lives as stressful. Responses range from 0 (never) to 4 (very often), yielding a total score between 0 and 40, with higher scores indicating greater perceived stress (Cohen et al. 1983).
- Generalized Anxiety Disorder‐7 (GAD‐7) GAD‐7 was a self‐report‐based survey that assesses generalized anxiety disorder and severity on a 7‐item scale. Scores of 5, 10, and 15 were taken as the cut‐off points for mild, moderate, and severe anxiety, respectively. The total score, ranging from 0 to 21, provided an indication of the severity of anxiety symptoms, with higher scores corresponding to greater symptom severity (Spitzer et al. 2006).
- Satisfaction with Life Scale (SWLS): The Satisfaction with Life Scale (SWLS) is a 5‐item self‐report measure designed to assess global cognitive judgments of one's overall life satisfaction. Each item was rated on a 7‐point Likert scale ranging from 1 (“strongly disagree”) to 7 (“strongly agree”). Higher scores indicate greater life satisfaction (Diener et al. 1985).
- Everyday Cognition 12 Scale (ECog‐12): The ECog‐12 is a validated, informant‐rated scale designed to evaluate changes in everyday cognitive functioning. Participants rate observed changes over the past decade using a 4‐point Likert scale. Respondents are asked to compare their current ability in these areas with their functioning from 10 years ago, ranging from 1 (better or no change) to 4 (consistently much worse). Higher scores indicate greater perceived functional cognitive decline (Farias et al. 2008).
- Dysfunctional Attitudes Scale‐17 (DAS‐17): The DAS‐17 is a shortened version of the original 40‐item Dysfunctional Attitudes Scale (DAS) and is designed to assess maladaptive beliefs and cognitive distortions associated with vulnerability to depression and psychological distress. Participants were asked to rate the degree to which they agreed or disagreed with each of the 17 total statements on a 7‐point Likert scale, typically ranging from 1 (fully disagree) to 7 (fully agree). The total score was the sum of the values for each individual item.
- Short Form 36 (SF‐36) The SF‐36 is a 36‐item questionnaire used to measure health‐related quality of life across eight health domains: physical functioning, role limitations due to physical health, role limitations due to emotional problems, vitality (energy/fatigue), emotional well‐being, social functioning, pain, and general health perceptions. Items were rated using Likert‐type scales, and all domain scores were transformed to a 0–100 scale, where higher scores indicated better health status and functioning (Ware and Sherbourne 1992).
- PERMA Profiler: Positive Emotion, Negative Emotion, Engagement, Relationships, Meaning, and Accomplishment profile. The PERMA Profiler is a comprehensive self‐reported instrument designed to measure average well‐being across five key dimensions as outlined in Martin Seligman's PERMA model: Positive Emotion, Engagement, Relationships, Meaning, and Accomplishment. Additionally, a single‐item question related to happiness is also measured (Butler and Kern 2016).
- Abbreviated POMS, as well as CNS Vital Signs, Cognitive Performance was evaluated as previously described (Grove and Prapavessis 1992).
- Adverse events (AEs) associated with daily supplementation and compliance were also recorded throughout the study period to assess the safety profile of supplementation.

### 2.3 Statistical Analysis

Given the exploratory nature of the studies, a prior sample size analysis was performed, where a minimum of 12–16 subjects were accepted for a preliminary observation. The statistical analysis of the first clinical study was designed to assess the significance of primary and secondary outcomes using generalized linear mixed models (GLMM). Specifically, in this analysis, changes over time (baseline, 60 min, 180 min, and Week 4) between the placebo and GBP480 groups were treated as a categorical repeated measures variable (within‐subject factor), while treatment groups (Placebo and GBP480) were treated as a between‐subjects factor. To account for individual variability, subjects were considered as a random factor. Pairwise comparisons of marginal means were analyzed using sequential Bonferroni correction, adjusting for multiple comparisons. This ensured rigorous statistical control. The model accounted for interactions between time and treatment group, with statistical significance established at *p* < 0.05 for all analyzes.

For the second study, to explore changes between and within groups, repeated measures ANOVA was calculated. Pairwise comparisons between means were done using a *t*‐test for independent or paired samples, depending on the situation. In the scope of the repeated measures ANOVA, Holm‐Bonferroni correction for multiple comparisons was used on the statistical significance of post hoc *t*‐tests. Cohen's d statistics corrected for correlation between repeated measures were used as the effect size measure for baseline‐4 weeks comparisons within study groups. Cohen's d statistics for between‐subject designs were used as effect sizes for comparing group means. Effect sizes using Cohen's d are described as trivial (0–0.19), small (0.2–0.49), moderate (0.5–0.79), and large (> 0.8). All statistical analyzes were performed using SPSS version 20 (IBM SPSS Inc., New York, NY, USA). To evaluate changes in categorical stress and anxiety levels over time within the GBP Doses, a chi‐squared analysis was conducted, followed by a Cramer's V for effect size differences.

## 3 Results

### 3.1 Baseline Characteristics and Demographics

As referenced previously, the first study was part of a broader investigation involving four total arms: GBP; grape seed extract, bilberry extract, and placebo (Kara et al. 2025). Only data from the GBP480 group and the placebo were analyzed. A total of 41 participants were enrolled in the first study and were randomized to receive either the extract supplement GBP480 (*n* = 21) or a placebo (*n* = 20). Of these enrolled participants, 16 were allocated to the GBP480 group, and 16 to the placebo group, who completed the study and were included in the final analysis. In the second study, 30 participants were screened and randomized (14 to GBP480, 16 to GBP240). A total of 28 participants completed the study and were included in the final analysis (13 in GBP480, 15 in GBP240). For both studies, participants who did not complete the study were lost to follow‐up or withdrew due to personal reasons or adverse events. All groups were assessed at baseline and again after 4 weeks.

**FIGURE 1:**
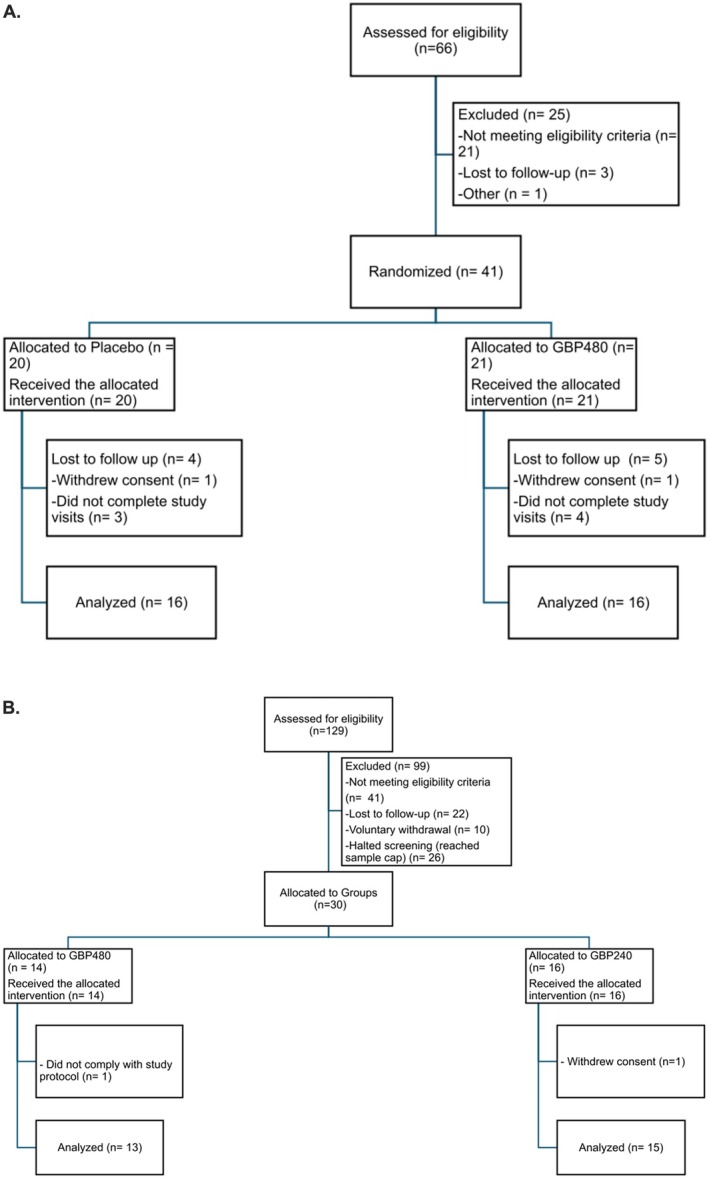
CONSORT flow diagram of two clinical studies: (A) First study RCT, GBP480 versus placebo; (B) Second study RCT versus two different GBP doses. 480: 480 mg daily; 240: 240 mg daily; GBP: *Ginkgo biloba* phosphatidylserine.

Baseline characteristics are shown in Table 1 and the CONSORT flow chart in Figure 1.

**TABLE 1:** Baseline characteristics and demographics of two clinical studies.

Characteristic	First study		Second study	
Supplementation	PLACEBO	GBP 480	GBP 480	GBP 240
Total (*n*)	16	16	13	15
Male (*n*, %)	7, 43.75	4, 25.0	7, 53.84	7, 46.67
Female (*n*, %)	9, 56.25	12, 75.0	6, 46.16	8, 53.33
Age (years)	38.63 ± 7.62	38.31 ± 7.54	58.38 ± 7.19	57.33 ± 4.70

*Note* *:* Age is expressed as mean ± standard deviation. Number of subjects and gender is count and percent of sample size.

Abbreviations: 480: 480 mg daily; 240: 240 mg daily; GBP: *Ginkgo biloba* phosphatidylserine.

No severe adverse events were reported in either of the studies. In the first study, no adverse events were reported in either the GBP480 or the placebo group, and no compliance issues were raised in both groups throughout the study period. In the second study, three adverse events were reported as nonsevere, all occurring in the GBP240 group by separate participants. Including a participant who reported transient blurred vision after 2 days' usage, and then chose to withdraw the informed consent and discontinue the product. The other two events were mild in severity and included temporary light‐headedness and occasional difficulty staying asleep. No action was taken in response to the latter two events. All events were considered possibly related to the study product. The symptoms for all subjects resolved over time. Given the lack of similar events in the GBP480 group and the absence of a consistent pattern, these findings do not indicate a dose‐dependent relationship and are interpreted as reflecting individual variability rather than a systematic effect of the intervention.

### 3.2 First Clinical Study: RTC Versus Placebo

#### 3.2.1 Cognitive Performance

**FIGURE 2:**
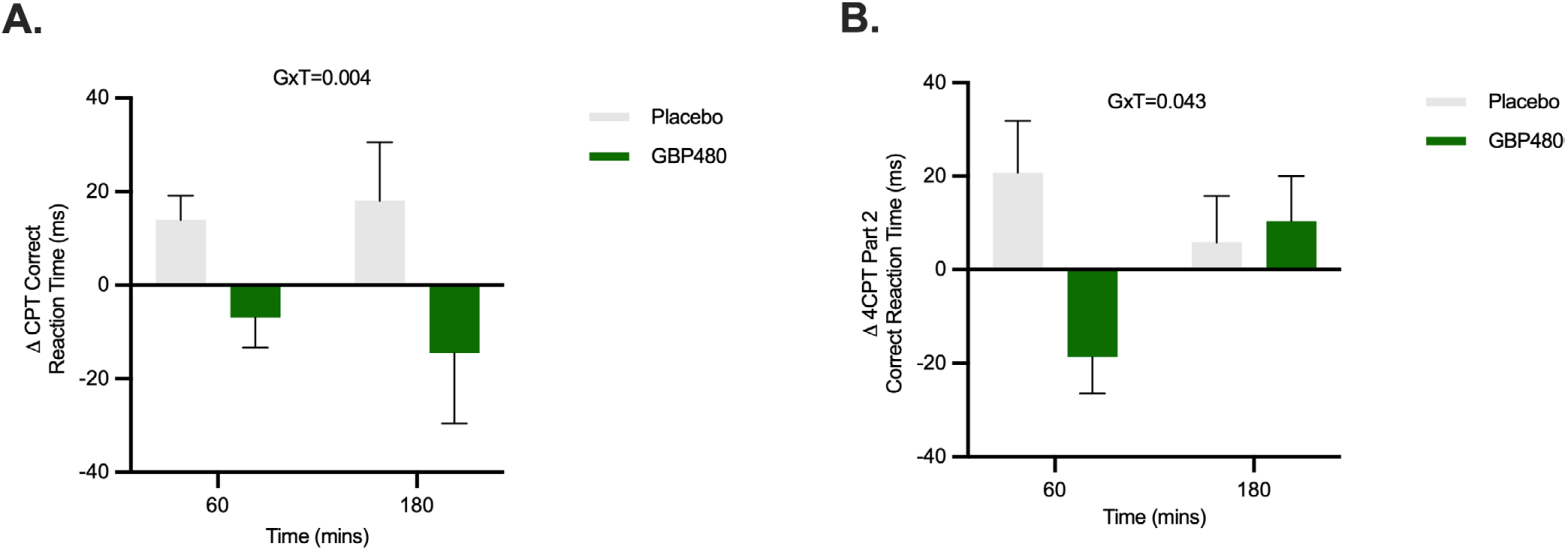
Delta changes for GBP acute (single dose) versus placebo on: (A) Continuous performance test and (B) 4PCPT part 2. (A) Significant group by time interaction was demonstrated for choice reaction time correct responses for the continuous performance test (*p = 0.004). (B) Delta changes for part 2 of the 4 part continuous performance test, acutely, a significant group by time interaction was detected for average reaction time correct responses (*p = 0.043). Values are reported as means ± SEM. 480: 480 mg daily; 4PCPT: Four‐part continuous performance test; Δ: Delta change; GBP: *Ginkgo biloba* phosphatidylserine; GxT: G‐squared statistics.

In the first study, when examining the acute (single dosage) effects of study treatment on neurocognitive assessments, we found a significant group by time interaction for Choice Reaction Time Correct Responses for the Continuous Performance Test (*p* = 0.004) and pairwise comparison showed that GBP480 was significantly higher than placebo at time Pre (*p* = 0.041, Placebo = 426.00 ± 45.81 versus GBP480 = 464.19 ± 57.62, *g* = 0.72). At subsequent time points, mean values in the supplement group decreased while those in the placebo increased, creating a nearly balanced mean at 180 min. When comparing the change from 60 min to Pre (*g* = −1.097) and 180 min to Pre between groups (*g* = −0.693), effect sizes were in favor of GBP480 (Figure 2A). However, pairwise comparisons showed no significant differences between or within groups. Furthermore, we did not observe consistent outcome across all measures of reaction time (data not shown).

**FIGURE 3:**
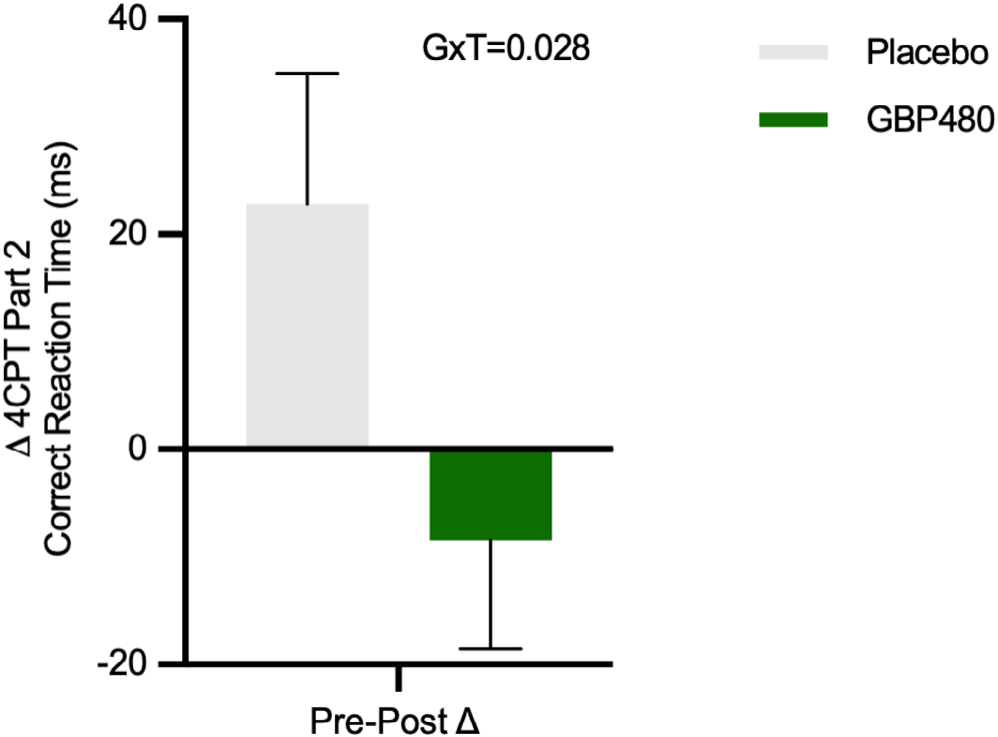
GBP 4‐week supplementation versus placebo. Delta change for part 2 of the 4 part continuous performance test, after 4 weeks, a significant group by time interaction was demonstrated for average reaction time correct responses (*p = 0.028). Values are reported as means ± SEM. 480: 480 mg daily; 4PCPT: Four‐part continuous performance test; Δ: Delta change; GBP: *Ginkgo biloba* phosphatidylserine; GxT: G‐squared statistics.

For the acute (single dose) responses to Part 2 of the 4 Part Continuous Performance Test (Figure 2B), a significant group by time interaction was detected for Average Reaction Time Correct Responses (*p* = 0.043); however, no significant between‐group differences were observed on pairwise comparisons. When examining the raw data, the Placebo has a small increase from Pre to 60 min according to effect size (*g* = 0.389), whereas GBP480 has a small decrease (*g* = −0.384), and at 180 min, both groups approach Pre values. Consistent with the acute findings, after 4‐weeks of supplementation, the 4PCPT—Part 2 showed a significant group*time interaction for Average Reaction time correct responses (*p* < 0.028, Figure 3), with Group C directionally going downward compared to baseline (mean diff = −8.44 ms, *g* = −0.293); and Placebo directionally going upward compared to baseline (Placebo: mean diff = 22.81 ms, *g* = 0.423).

**FIGURE 4:**
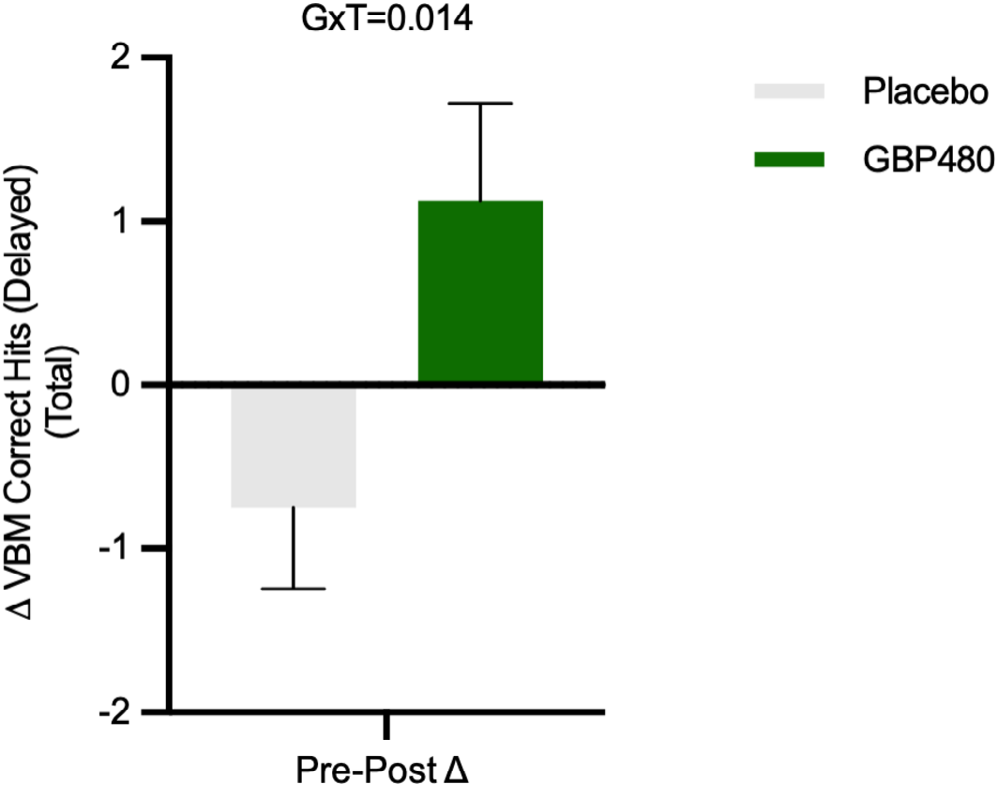
GBP 4‐week supplementation vs. placebo on verbal memory (VBM) test. Total correct hits (delayed) after 4 weeks, a significant group by time interaction was detected for correct hits delayed (*p = 0.014). Values are reported as means ± means standard error (SEM). 480: 480 mg daily; Δ: Delta change; GBP: *Ginkgo biloba* phosphatidylserine; GxT: G‐squared statistics.

Regarding verbal memory, after 4‐week supplementation we found a significant group * time interaction for correct hits delayed on the Verbal Memory Test indicating that pre‐ to postchange was significantly different between groups (*p* = 0.014; Group C: mean diff = 1.22 hits, *g* = 0.397; Placebo: mean diff = −0.75 hits, *g* = 0.021). Directionally, the immediate correct hits also tended to favor GBP, but this did not reach a statistically significant interaction. Overall, this suggests a directional response that is consistent with improved memory in GBP480, by scoring higher on total correct hits delayed during the Verbal Memory test (*p* < 0.05 in delayed, Figure 4).

#### 3.2.2 Mood States

The one significant effect on POMS was Depression and effect size analysis shows that GBP480 reported a moderate increase from Pre to 180 min after a single dosage in respect to placebo. No other significant group by time interactions were demonstrated.

### 3.3 Second Clinical Study: RCT Versus Two Doses

#### 3.3.1 Perceived Stress Scale (PSS‐10)

**FIGURE 5:**
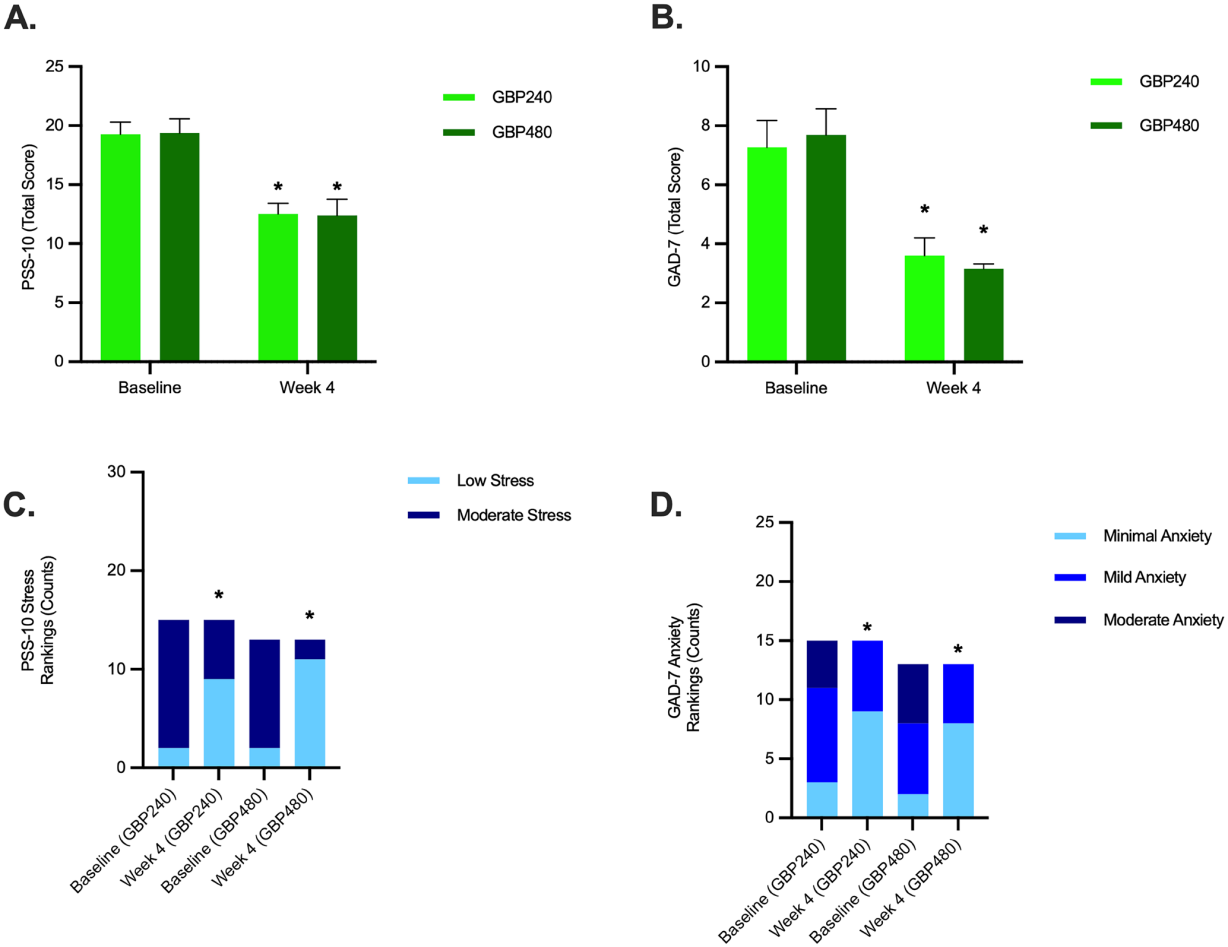
GBP 4‐week supplementation at different doses. (A) PSS‐10 total stress score, (B) GAD‐7 total anxiety score. (C) PSS‐10 stress rankings, and (D) GAD‐7 anxiety rankings. Values are reported as means ± SEM. *p < 0.05 vs. baseline. 480: 480 mg daily; 240: 240 mg daily; GAD‐7: Generalize Anxiety Disorder; GBP: *Ginkgo biloba* phosphatidylserine; PSS‐10: Perceived Stress Scale.

Results for changes in stress responses using PSS‐10 scale indicated very large and statistically significant reductions in stress from baseline to 4‐weeks in both groups, GBP480 (*p* < 0.001, −36.11%, *d* = 1.492), and GBP240 groups (*p* < 0.001, −34.95%, *d* = 1.787), with no significant differences between groups (Figure 5A, Table S1). Furthermore, to evaluate changes in categorical stress levels over time within the GBP Doses, a chi‐squared analysis was conducted, followed by a Cramer's V for effect size differences (Figure 5C). Results demonstrated significant and large changes in stress categories over time in both GBP480 (*p* < 0.001, V = 0.692), and GBP240 (*p* = 0.008, V = 0.484), with no differences between groups. Demonstrating a distinct shift in states of moderate stress at baseline, to low stress at 4‐weeks.

#### 3.3.2 Generalize Anxiety Disorder (GAD‐7)

Results in both groups exhibited strong statistical reductions in GAD‐7 total scores from baseline to Week 4. These reductions corresponded to large within‐group effect sizes, reflecting a meaningful reduction in self‐reported anxiety symptoms over the 4‐week period (Figure 5B, Table S1).

Similar results were seen for pairwise differences within treatment conditions in anxiety resilience; however, this was only trending from baseline to 4‐weeks in GBP480 (*p* = 0.079, −66.67%, *d* = 0.902), and GBP240 (*p* = 0.073, −43.75%, *d* = 0.912) (Table S1). To evaluate changes in categorical anxiety levels over time within the GBP doses, a chi‐squared analysis was conducted, followed by a Cramer's V for effect size differences (Figure 5D). Results showed significant and large changes in anxiety classifications over time in both GBP480 (*p* = 0.013, V = 0.578) and GBP240 (*p* = 0.026, V = 0.493), with no differences between groups, demonstrating a distinct shift in states of moderate anxiety at baseline, to mild to minimal anxiety at 4‐weeks.

#### 3.3.3 Satisfaction With Life Scale (SWLS)

The SWLS was used to evaluate subjective global life satisfaction as an indicator of well‐being. A statistically significant increase in SWLS scores was observed only in the GBP480 group (*p* = 0.001,38.43%, *d* = −1.030). No significant between‐group differences were observed. These findings indicate that both doses of GBP may contribute to enhancing subjective life satisfaction over time, with a more robust effect observed in the higher dose group (Table S1).

#### 3.3.4 Everyday Cognition 12 Scale (ECog‐12)

Scores on the ECog‐12 decreased directionally with a trend toward significance in the GBP480 group (*p* = 0.128, *d* = 0.650), and only directionally in the GBP240 group (*p* = 0.270, *d* = 0.498) over the course of the 4‐week testing period, indicating a possible improvement in perceived cognitive functioning. However, these changes did not reach statistical significance in either group, and there were no significant differences between groups (Table S1).

#### 3.3.5 Dysfunctional Attitudes Scale‐17 (DAS‐17)

Both groups showed directional decreases in total DAS‐17 scores from baseline to Week 4, with GBP240 demonstrating a statistically moderate reduction (*p* = 0.013, −17.10%, *d* = 0.448), while GBP480 did not reach statistical significance. The more robust response observed in GBP240 may be partly explained by higher baseline DAS‐17 scores, which converged with the GBP480 by Week 4.

Similarly, the perfectionism subscale showed a statistically significant decrease only in GBP240 (*p* = 0.014, −20.51%, *d* = 0.493) and no significant changes between timepoints or groups were observed for the dependency subscale (Table S1).

#### 3.3.6 Quality of Life

The Short Form 36 (SF‐36) is a widely used measure assessing overall health‐related quality of life across multiple domains including physical functioning, emotional well‐being, and social functioning. Results are summarized below (Table S2):

- *Energy/Fatigue subscale*: Both the GBP480 (*p* < 0.001, 63.16%, *d* = −1.689) and the GBP240 group (*p* = 0.001, 39.86%, *d* = −1.118) experienced large increases from baseline to 4 weeks.
- *Emotional Well‐being subscales*: Values of both groups increased strongly from baseline to 4 weeks with the GBP480 group also having a visually stronger increase (*p* < 0.001, 35.68%, *d* = −1.631) compared to the GBP240 group (*p* = 0.025, 17.58%, *d* = −0.896).
- Social Functioning subscale with values of both groups increased strongly from baseline to 4 weeks for the GBP480 group (*p* = 0.003, 25.00%, *d* = −1.128) and the GBP240 group (*p* = 0.019, 17.63%, *d* = 0.825) with no significant differences between groups.
- *General Health Perception*: Increased similarly in both groups, reaching marginal statistical significance only in GBP240 (*p* = 0.052, 14.74%, *d* = −0.494) compared to the GBP480 group (*p* = 0.304, 11.32%, *d* = −0.366) with no significant between‐group differences (Table S2). Lastly, neither GBP480 nor GBP240 groups showed significant improvements in physical function, physical health limitations, emotional health limitations, or pain.

#### 3.3.7 Emotional States (PERMA)

**FIGURE 6:**
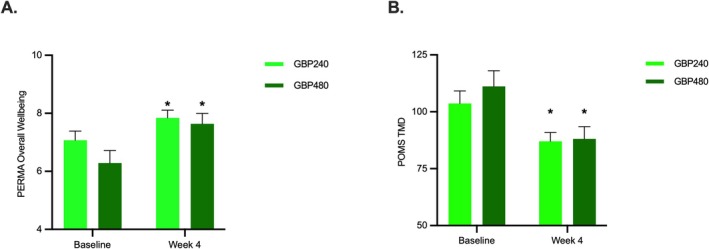
GBP 4‐week supplementation at different doses: (A) PERMA overall wellbeing (B) POMS (profile of mood states) TMD (total mood disturbance). Values are reported as means ± SEM. *p < 0.05 versus baseline. 480: 480 mg daily; 240: 240 mg daily; GBP: *Ginkgo biloba* phosphatidylserine; PERMA: Positive emotion, negative emotion, engagement, relationships, meaning, and accomplishment profile.

Results for changes in emotional responses using PERMA assessment scale indicated very large and statistically significant increases in positive emotional state from baseline to 4‐weeks in both GBP480 (*p* < 0.001, 26.55%, *d* = −0.959) and GBP240 (*p* = 0.003, 18.43%, *d* = −0.748), with no significant differences between groups. Similarly, the PERMA Overall subscale saw large to moderate increases in the GBP480 group (*p* < 0.001, 21.43%, *d* = −1.057) and the GBP240 group (*p* = 0.046, 10.89%, *d* = −0.604) from baseline to Week 4 (Figure 6A). Additionally, the GBP480 group experienced significant PERMA improvements for the Relationships (*p* < 0.001, 24.25%, *d* = −0.921), Meaning (*p* = 0.002, 20.50%, *d* = −0.997), and Accomplishments (*p* = 0.023, 18.70%, *d* = −0.721) sub‐domains, while the GBP240 group did not see significant changes from baseline to 4‐weeks in these domains. No significant treatment effects were found for either group over time for Engagement, Negative Emotions, Health, or Loneliness. However, a trend was observed in Negative Emotions for GBP240 (*p* = 0.060, −30.41%, *d* = 0.672). Overall, while no significant differences were observed between groups, visual trends suggest that GBP480 generally outperformed GBP240 across these well‐being measures (Table S3).

#### 3.3.8 Abbreviated Profile of Mood States (POMS)

The Abbreviated Profile of Mood States (POMS) assessed mood changes across multiple dimensions.

Tension decreased similarly in both groups, reaching statistical significance only in the GBP240 group (*p* = 0.025, −43.43%, *d* = 0.831), while GBP480 showed only a marginal increase (*p* = 0.099, −41.33%, *d* = 0.961). Anger tended to decrease directionally in both groups, particularly in GBP480, while fatigue experienced a strong and significant decrease in both GBP480 (*p* < 0.001, −59.07%, *d* = 1.287) and GBP240 (*p* = 0.013, −48.22%), with a slightly greater visual effect in GBP480. Both groups showed a strong shift toward decreased levels of depression when compared to baseline, although the changes were marginally statistically significant in GBP480 (*p* = 0.064, −56.67%, *d* = 0.731), as well as in GBP240 (*p* = 0.059, −59.38%, *d* = 0.731).

Specifically for esteem related affect in the GBP480 group, it was seen to have a significant within group effect when compared to baseline (*p* = 0.010, 26.74%, *d* = −0.873). GBP240 moderately increased for esteem, but the difference between time points was not statistically significant (*p* = 0.168, 13.16%, *d* = 0.494). Mean results for vigor significantly increased in GBP480 (*p* = 0.002, 63.51%, *d* = −0.912) and GBP240 (*p* = 0.038, 30.70%, *d* = −0.588), with a visually more pronounced increase in GBP480 (though not statistically different between groups). Confusion decreased strongly in both groups with GBP480 showing a slightly larger visual decrease compared to baseline (*p* = 0.006, −51.26%, *d* = 0.883) and more marginal in the GBP240 group (*p* = 0.011, −41.10%, *d* = 0.753).

Finally, both groups experienced large and statistically significant decreases in Total Mood Disturbance (TMD) in both GBP480 (*p* = 0.001, −20.76%, *d* = 1.134) and GBP240 (*p* = 0.014, −16.00.819) groups (Figure 6B, Table S4). Higher TMD scores reflect greater overall mood disturbance, while lower scores indicate a more positive mood profile suggesting that GBP treatments reduced overall mood disturbances in subjects.

Overall, for the POMS domains, both groups significantly improved various mood states over time. While no significant between‐group differences were observed, some visual trends appeared to favor GBP480.

#### 3.3.9 Cognitive Domains

Analysis of cognitive domains revealed no statistically significant differences in either GBP480 or GBP240 groups over time (data not shown). Cognitive performance outcomes frequently show variability and small effect sizes that may reflect random variation rather than true treatment effects (CNS Vital Signs 2025).

#### 3.3.10 Cognitive Assessments

Similarly, no significant group differences emerged across the computerized cognitive assessments. Both groups demonstrated a directional slowing in complex reaction time on the Stroop test. However, the increase in GBP240 was moderate to large and statistically significant (*p* = 0.004, 9.91%, *d* = −0.692). While the increase in GBP480 (*p* = 1.000, 2.95%, *d* = −0.165) was slight and nonsignificant. Interestingly, reaction times across all four tasks of the Four‐Part Continuous Performance Test (4PCPT) showed consistent directional decreases in both groups, but these changes did not reach statistical significance and were small in magnitude.

## 4 Discussion

These trials investigated the efficacy of a combination of standardized *Ginkgo biloba* extract (GB) and phosphatidylserine (Virtiva Plus, GBP), in supporting cognitive function, mood regulation, and overall quality of life. The formula demonstrated promising preliminary evidence on emotional balance and cognitive performance, possibly linked to neuroprotective properties, likely mediated through enhanced microcirculation and central nervous system support (Kara et al. 2026). Thus, microcirculation, defined as blood flow through the smallest vessels, is essential for optimal brain function. Its impairment is linked to cognitive decline, while its maintenance supports memory, attention, and general cognitive performance (De Silva and Faraci 2016).

The most consistent and noteworthy findings in relation to cognition emerged in the first study, in which, following both acute (single dose) and chronic (4‐week) supplementation, participants receiving GBP exhibited improvements in delayed verbal memory (correct hits) and reaction time, suggesting a possible beneficial impact on cognitive functions. However, this study was limited by its relatively small sample size, which likely reduced statistical power. Additionally, the study population consisted of healthy adults who may exhibit less sensitivity compared to individuals with mild cognitive impairment or clinical affective disorders (Jongsiriyanyong and Limpawattana 2018). Such populations tend to respond more strongly to cognitive or mood‐enhancing treatments, suggesting that further research in these groups may be warranted.

The second study focused on stress and anxiety outcomes, in participants with elevated stress levels. Chronic stress, low mood, and depressive states in adulthood are linked to a range of negative outcomes, including anxiety, substance use, poor professional performance, and risky behaviors. While pharmacological treatments have advanced, many individuals experience limited relief, unwanted side effects, or delayed benefits. As a result, there is growing interest in alternative approaches, particularly brain health supplements. *Ginkgo biloba* is one such option, recognized for its potential to support mood and cognitive function through antioxidant, anti‐inflammatory, and circulation‐enhancing effects; however, its effectiveness has been limited by poor bioavailability (Singh et al. 2019; Wang et al. 2022). Phosphatidylserine has shown to provide a significant benefit for cognitive functions including memory, learning, vocabulary skills, and concentration (Dong et al. 2007). To address this, the current study examined the effects of GBP in participants experiencing heightened stress. The overall findings demonstrated that GBP, the synergistic association of a highly standardized GB extract and phosphatidylserine, significantly reduced perceived stress and anxiety in individuals with elevated baseline stress levels, regardless of dosage. The ingredient was also able to improve various mood states, and the results further demonstrated that these effects tended to be more pronounced at a full dosage of GBP (GBP at 480 mg/day), suggesting a potential dose‐dependent response on certain mood domains, albeit no statistically significant between‐group differences were observed. These results align with existing evidence according to a literature review of similar published studies. The anxiolytic properties of *Ginkgo biloba* are well‐supported, with clinical trials demonstrating significant reductions in anxiety symptoms, potentially through mechanisms involving antioxidant activity and modulation of neurotransmitter systems (Woelk et al. 2007).

Cognitive outcomes were largely null across the duration of the second study and showed modest and inconsistent changes overall. However, a visually downward trend in reaction times across all four cognitive performance tests was observed at both treatment dosages, which could suggest improved processing speed in response to various stimuli. Although the changes were small and not statistically significant in this study, the improvement across multiple reaction time measures suggests a subtle effect. This pattern aligns with previous findings and may reach significance with a larger sample, control group, or longer treatment duration. Thus, despite the promising findings, while both dosing groups (240 vs. 480 mg daily) showed directional improvements across numerous outcomes, without a control arm, the true magnitude of supplement effects remains unclear. Additionally, the short duration of this study is another important consideration. While most *Ginkgo biloba* studies spanned 12 weeks or more, meaningful improvements were observed in our trials after just 4 weeks. This suggested potential for early benefits on stress modulation, but longer trials are needed to evaluate the stability and durability of any clear cognitive effects over time. Overall, the findings of the second dose‐related study produced significant reductions in perceived stress and generalized anxiety symptoms over a 4‐week period in adults with elevated baseline stress levels, supporting the emotional balance and life satisfaction potentials of this dietary supplement. While the 480‐dose group generally exhibited numerically greater improvements in mood measures, statistical comparisons between doses did not reveal significant differences, suggesting that the lower dose confers substantial benefits, which are comparable to the high dosage. In contrast, cognitive performance outcomes were modest and not definitive in this population, suggesting that cognitive benefits may be more readily observable in healthy adults or under different experimental conditions. Nonetheless, given the growing prevalence and societal impact of stress‐related disorders, findings from both studies underscore the potential of GBP as a natural, well‐tolerated tool for emotional well‐being and cognitive support.

The promising, even if exploratory, results described in the present research confirmed previous reported advantages of associating *Gingko biloba* and phosphatidylserine in a unique supplement, so combining antioxidant, neuroprotective and nootropic properties of both components. Further research is needed to consolidate this preliminary evidence.

## 5 Conclusions

Collectively, these research studies support the findings that GBP is a well‐tolerated, natural tool for supporting cognitive function, mood, life satisfaction, and alleviating symptoms of stress and anxiety, especially under conditions of heightened stress. This formulation may serve as a complementary option to conventional treatments, particularly for individuals seeking alternatives to pharmacologic therapies that may be associated with delayed onset of action or undesirable side effects. Although results are preliminary, they are encouraging and coherent with the proposed mechanisms of action, namely, neuroprotection, antioxidant effects, and support for cerebral microcirculation.

Further research involving larger, more diverse populations and extended supplementation periods is necessary to confirm these outcomes and refine the optimal dosing strategy. Nonetheless, the observed improvements in psychological well‐being and cognitive resilience support the potential of the combination of this highly standardized *Ginkgo biloba* extract with phosphatidylserine, Virtiva Plus, for brain health and emotional balance.

## Author Contributions

**Justine Davis:** methodology, writing – review and editing, data curation, formal analysis. **Ryan Lowery:** conceptualization, methodology, writing – review and editing. **Giovanna Petrangolini:** conceptualization, writing – review and editing. **Gabriel Wilson:** conceptualization, methodology, data curation, formal analysis, writing – review and editing, validation. **Kenny Hawkins:** conceptualization, methodology, data curation, formal analysis, writing – review and editing. **Cristal Abreu Matias:** writing – review and editing, data curation. **Jacob Wilson:** conceptualization, methodology, data curation, validation, formal analysis, supervision, writing – review and editing, project administration. **Paola Misiano:** writing – original draft, validation.

## Ethics Statement

The clinical Protocols, First Study (ID Pro00074459) and Second Study (ID Pro00081043) were approved by an external Institutional Review Board, Advarra IRB (USA), in September 2023 and August 2024, respectively, and were conducted in compliance with the Declaration of Helsinki and were registered at ClinicalTrials.gov under registration number NCT06309914 (First Study) and NCT06583941 (Second Study).

## Consent

Informed consent was obtained from all subjects involved in the study.

## Conflicts of Interest

G.P. is an employee of Indena S.p.A., Milan, Italy. Virtiva is a trademark owned by Indena S.p.A., Italy. The other authors declare no conflicts of interest.

## Supporting information


**Table S1:** Descriptive statistics: PSS‐10, GAD‐7, SWLS, Ecog‐12, and DAS‐17 Surveys.
**Table S2:** Descriptive statistics: SF‐36.
**Table S3:** Descriptive statistics: PERMA.
**Table S4:** Descriptive statistics: POMS.

## Data Availability

The data that support the findings of this study are available on request from the corresponding author. The data are not publicly available due to privacy or ethical restrictions.

## References

[fsn372001-bib-0001] Achete de Souza, G. , S. V. de Marqui , J. N. Matias , E. L. Guiguer , and S. M. Barbalho . 2020. “Effects of *Ginkgo biloba* on Diseases Related to Oxidative Stress.” Planta Medica 86, no. 6: 376–386. 32097975 10.1055/a-1109-3405

[fsn372001-bib-0002] Akanchise, T. , and A. Angelova . 2023. “ *Ginkgo biloba* and Long COVID: In Vivo and in Vitro Models for the Evaluation of Nanotherapeutic Efficacy.” Pharmaceutics 15, no. 5: 1562. 37242804 10.3390/pharmaceutics15051562 PMC10224264

[fsn372001-bib-0003] Barbalho, S. M. , R. Direito , L. F. Laurindo , et al. 2022. “Gingko Biloba in the Aging Process: A Narrative Review.” Antioxidants 11, no. 3: 525. 35326176 10.3390/antiox11030525 PMC8944638

[fsn372001-bib-0004] Brondino, N. , A. De Silvestri , S. Re , et al. 2013. “A Systematic Review and Meta‐Analysis of *Ginkgo biloba* in Neuropsychiatric Disorders: From Ancient Tradition to Modern‐Day Medicine.” Evidence‐Based Complementary and Alternative Medicine 2013: 915691. 23781271 10.1155/2013/915691 PMC3679686

[fsn372001-bib-0005] Butler, J. , and M. L. Kern . 2016. “The PERMA‐Profiler: A Brief Multidimensional Measure of Flourishing.” International Journal of Wellbeing 6, no. 3: 1–48.

[fsn372001-bib-0006] Caglayan, C. , P. Taslimi , C. Türk , F. M. Kandemir , Y. Demir , and İ. Gulcin . 2019. “Purification and Characterization of the Carbonic Anhydrase Enzyme From Horse Mackerel ( *Trachurus trachurus* ) Muscle and the Impact of Some Metal Ions and Pesticides on Enzyme Activity.” Comparative Biochemistry and Physiology. Toxicology & Pharmacology 226: 108605. 31422160 10.1016/j.cbpc.2019.108605

[fsn372001-bib-0007] Cheung, F. , Y. L. Siow , and W. Z. Chen . 1999. “Inhibitory Effect of *Ginkgo biloba* Extract on the Expression of Inducible Nitric Oxide Synthase in Endothelial Cells.” Biochemical Pharmacology 58, no. 10: 1665–1673. 10535759 10.1016/s0006-2952(99)00255-5

[fsn372001-bib-0008] CNS Vital Signs . 2025. “CNS Vital Signs Brief Interpretation Guide”. https://www.cnsvs.com/WhitePapers/CNSVS‐BriefInterpetrationGuide.pdf.

[fsn372001-bib-0009] Cohen, S. , T. Kamarck , and R. Mermelstein . 1983. “A Global Measure of Perceived Stress.” Journal of Health and Social Behavior 24, no. 4: 385–396. 6668417

[fsn372001-bib-0010] De Silva, T. M. , and F. M. Faraci . 2016. “Microvascular Dysfunction and Cognitive Impairment.” Cellular and Molecular Neurobiology 36, no. 2: 241–258. 26988697 10.1007/s10571-015-0308-1 PMC4846472

[fsn372001-bib-0011] DeFeudis, F. V. 1991. *Ginkgo biloba* Extract (EGb 761): Pharmacological Activities and Clinical Applications. Elsevier.

[fsn372001-bib-0012] Di Pierro, F. , S. Togni , F. Franceschi , R. Eghenhofner , and L. Giacomelli . 2016. “Effects of Standardized *Ginkgo biloba* Extract Complexed With Phosphatidylserine (Virtiva) on Physiological Response to Prolonged, Intense Physical Activity.” Minerva Ortopedica e Traumatologica 67, no. 3: 119–123.

[fsn372001-bib-0013] Diener, E. , R. A. Emmons , R. J. Larsen , and S. Griffin . 1985. “The Satisfaction With Life Scale.” Journal of Personality Assessment 49, no. 1: 71–75. 16367493 10.1207/s15327752jpa4901_13

[fsn372001-bib-0014] Dong, X. X. , Z. J. Hui , W. X. Xiang , Z. F. Rong , S. Jian , and C. J. Zhu . 2007. “ *Ginkgo biloba* Extract Reduces Endothelial Progenitor‐Cell Senescence Through Augmentation of Telomerase Activity.” Journal of Cardiovascular Pharmacology 49, no. 2: 111–115. 17312453 10.1097/FJC.0b013e31802ef519

[fsn372001-bib-0015] Duffy, M. E. , J. M. Twenge , and T. E. Joiner . 2019. “Trends in Mood and Anxiety Symptoms and Suicide‐Related Outcomes Among U.S. Undergraduates, 2007–2018: Evidence From Two National Surveys.” Journal of Adolescent Health 65, no. 5: 590–598. 10.1016/j.jadohealth.2019.04.033 31279724

[fsn372001-bib-0016] Farias, S. T. , D. Mungas , B. R. Reed , et al. 2008. “The Measurement of Everyday Cognition (ECog): Scale Development and Psychometric Properties.” Neuropsychology 22, no. 4: 531–544. 18590364 10.1037/0894-4105.22.4.531 PMC2877034

[fsn372001-bib-0017] Field, B. H. , and R. Vadnal . 1998. “ *Ginkgo biloba* and Memory: An Overview.” Nutritional Neuroscience 1, no. 4: 255–267. 27414695 10.1080/1028415X.1998.11747236

[fsn372001-bib-0018] Franke, A. G. , I. Heinrich , K. Lieb , and A. Fellgiebel . 2014. “The Use of *Ginkgo biloba* in Healthy Elderly.” Age (Dordrecht, Netherlands) 36, no. 1: 435–444. 23736956 10.1007/s11357-013-9550-y PMC3889903

[fsn372001-bib-0019] Glade, M. J. , and K. Smith . 2015. “Phosphatidylserine and the Human Brain.” Nutrition 31, no. 6: 781–786. 25933483 10.1016/j.nut.2014.10.014

[fsn372001-bib-0020] Grove, B. , and H. Prapavessis . 1992. “Preliminary Evidence for the Reliability and Validity of an Abbreviated Profile of Mood States.” International Journal of Sport Psychology 23: 93–109.

[fsn372001-bib-0021] Hoff, E. , D. Zou , S. Schiza , et al. 2020. “Carbonic Anhydrase, Obstructive Sleep Apnea and Hypertension: Effects of Intervention.” Journal of Sleep Research 29, no. 2: e12956. 31808986 10.1111/jsr.12956

[fsn372001-bib-0022] Jongsiriyanyong, S. , and P. Limpawattana . 2018. “Mild Cognitive Impairment in Clinical Practice: A Review Article.” American Journal of Alzheimer's Disease and Other Dementias 33, no. 8: 500–507. 10.1177/1533317518791401 PMC10852498 30068225

[fsn372001-bib-0023] Kara, M. , G. Hasbal‐Celikok , P. Gómez‐Serranillos , et al. 2026. “In Vitro Studies to Investigate the Potential Neuroprotective and Neurotransmitter Modulation Effects of a Standardized *Ginkgo biloba* Extract Associated With Phosphatidylserine.” Frontiers in Nutrition 13: 1764334. 41788686 10.3389/fnut.2026.1764334 PMC12958353

[fsn372001-bib-0024] Kara, M. , G. Hasbal‐Celikok , J. Wilson , et al. 2025. “In Vitro Mechanistic Studies and Potential Health Benefits of a Standardized Bilberry Extract in Low Mood and Cognitive Enhancement.” Frontiers in Nutrition 12: 1630147. 40823035 10.3389/fnut.2025.1630147 PMC12352346

[fsn372001-bib-0025] Kasapoğlu, Ö. , A. Çakici , R. Sağlamtaş , et al. 2026. “Bioactive Potential of *Hippophae rhamnoides* L. Extracts: Comparative Antioxidant, Antimicrobial, and Enzyme Inhibitory Effects.” ChemistrySelect 11, no. 3: e04774.

[fsn372001-bib-0026] Kennedy, D. O. , C. F. Haskell , P. L. Mauri , and A. B. Scholey . 2007. “Acute Cognitive Effects of Standardised *Ginkgo biloba* Extract Complexed With Phosphatidylserine.” Human Psychopharmacology 22, no. 4: 199–210. 17457961 10.1002/hup.837

[fsn372001-bib-0027] Kim, H. Y. , B. X. Huang , and A. A. Spector . 2014. “Phosphatidylserine in the Brain: Metabolism and Function.” Progress in Lipid Research 56: 1–18. 24992464 10.1016/j.plipres.2014.06.002 PMC4258547

[fsn372001-bib-0028] Li, D. , J. Ma , B. Wei , S. Gao , Y. Lang , and X. Wan . 2023. “Effectiveness and Safety of *Ginkgo biloba* Preparations in the Treatment of Alzheimer's Disease: A Systematic Review and Meta‐Analysis.” Frontiers in Aging Neuroscience 15: 1124710. 36960422 10.3389/fnagi.2023.1124710 PMC10028084

[fsn372001-bib-0029] Lotharius, J. , F. J. Gamo‐Benito , I. Angulo‐Barturen , et al. 2014. “Repositioning: The Fast Track to New Anti‐Malarial Medicines?” Malaria Journal 13: 143. 24731288 10.1186/1475-2875-13-143 PMC4021201

[fsn372001-bib-0030] Ma, X. , X. Li , W. Wang , M. Zhang , B. Yang , and Z. Miao . 2022. “Phosphatidylserine, Inflammation, and Central Nervous System Diseases.” Frontiers in Aging Neuroscience 14: 975176. 35992593 10.3389/fnagi.2022.975176 PMC9382310

[fsn372001-bib-0031] Muscaritoli, M. 2021. “The Impact of Nutrients on Mental Health and Well‐Being: Insights From the Literature.” Frontiers in Nutrition 8: 656290. 33763446 10.3389/fnut.2021.656290 PMC7982519

[fsn372001-bib-0032] Özdemir, K. , and Y. Demir . 2025. “Phenolic Compounds in Exercise Physiology: Dual Role in Oxidative Stress and Recovery Adaptation.” Food Science & Nutrition 13, no. 8: e70714. 40735406 10.1002/fsn3.70714 PMC12301576

[fsn372001-bib-0033] Pagotto, G. , L. M. O. D. Santos , N. Osman , et al. 2024. “Gingko Biloba: A Leaf of Hope in the Fight Against Alzheimer's Dementia: Clinical Trial Systematic Review.” Antioxidants 13, no. 6: 651. 38929090 10.3390/antiox13060651 PMC11201198

[fsn372001-bib-0034] Silberstein, R. B. , A. Pipingas , J. Song , D. A. Camfield , P. J. Nathan , and C. Stough . 2011. “Examining Brain‐Cognition Effects of *Ginkgo biloba* Extract: Brain Activation in the Left Temporal and Left Prefrontal Cortex in an Object Working Memory Task.” Evidence‐Based Complementary and Alternative Medicine 2011: 164139. 21941584 10.1155/2011/164139 PMC3166615

[fsn372001-bib-0035] Singh, S. K. , S. Srivastav , R. J. Castellani , G. Plascencia‐Villa , and G. Perry . 2019. “Neuroprotective and Antioxidant Effect of *Ginkgo biloba* Extract Against AD and Other Neurological Disorders.” Neurotherapeutics 16, no. 3: 666–674. 31376068 10.1007/s13311-019-00767-8 PMC6694352

[fsn372001-bib-0036] Spitzer, R. L. , K. Kroenke , J. B. Williams , and B. Löwe . 2006. “A Brief Measure for Assessing Generalized Anxiety Disorder: The GAD‐7.” Archives of Internal Medicine 166, no. 10: 1092–1097. 16717171 10.1001/archinte.166.10.1092

[fsn372001-bib-0037] Tomino, C. , S. Ilari , V. Solfrizzi , et al. 2021. “Mild Cognitive Impairment and Mild Dementia: The Role of Gingko Biloba (EGb 761).” Pharmaceuticals 14, no. 4: 305. 33915701 10.3390/ph14040305 PMC8065464

[fsn372001-bib-0038] Unger, M. 2013. “Pharmacokinetic Drug Interactions Involving *Ginkgo biloba* .” Drug Metabolism Reviews 45, no. 3: 353–385. 23865865 10.3109/03602532.2013.815200

[fsn372001-bib-0039] Wang, X. , A. A. Memon , K. Palmér , A. Hedelius , J. Sundquist , and K. Sundquist . 2022. “Role of Multiple Risk Factors in Mental Disorders Diagnosed in Middle‐Aged Women: A Population‐Based Follow‐Up Study.” Journal of Psychiatric Research 156: 414–421. 36323144 10.1016/j.jpsychires.2022.10.040

[fsn372001-bib-0040] Ware, J. E. , and C. D. Sherbourne . 1992. “The MOS 36‐Item Short‐Form Health Survey (SF‐36). I. Conceptual Framework and Item Selection.” Medical Care 30, no. 6: 473–483. 1593914

[fsn372001-bib-0041] Woelk, H. , K. H. Arnoldt , M. Kieser , and R. Hoerr . 2007. “ *Ginkgo biloba* Special Extract EGb 761 in Generalized Anxiety Disorder and Adjustment Disorder With Anxious Mood: A Randomized, Double‐Blind, Placebo‐Controlled Trial.” Journal of Psychiatric Research 41, no. 6: 472–480. 16808927 10.1016/j.jpsychires.2006.05.004

[fsn372001-bib-0042] Zhang, H. F. , L. B. Huang , Y. B. Zhong , et al. 2016. “An Overview of Systematic Reviews of of *Ginkgo biloba* Extracts for Mild Cognitive Impairment and Dementia.” Frontiers in Aging Neuroscience 8: 276. 27999539 10.3389/fnagi.2016.00276 PMC5138224

[fsn372001-bib-0043] Zor, M. , R. Sağlamtaş , A. Demirci , E. Ceyran , K. Fettahoğlu , and Y. Demir . 2025. “Chemical Composition, Antimicrobial, and Enzyme Inhibitory Properties of *Elaeagnus angustifolia* L.” Chemical Biology & Diversity 22, no. 5: e202402802. 10.1002/cbdv.202402802 39744888

