# Supplementary material for: Health Benefits of a Standardized Ginkgo biloba Extract Associated With Phosphatidylserine in Alleviating Mental Stress and Cognitive Performance: Results From Two Exploratory Randomized Trials
Source: Food Sci Nutr. 2026 Jun 11;14(6):e72001. doi: 10.1002/fsn3.72001 (PMC13260694; doi:10.1002/fsn3.72001)
Supplement: Supplementary file 1 — Table S1: Descriptive statistics: PSS‐10, GAD‐7, SWLS, Ecog‐12, and DAS‐17 Surveys. Table S2: Descriptive statistics: SF‐36. Table S3: Descriptive statistics: PERMA. Table S4: Descriptive statistics: POMS. [file FSN3-14-e72001-s001.docx]

Supplementary Material for FOOD SCIENCE AND NUTRITION

**Supplementary Table 1S.** Descriptive Statistics: PSS-10, GAD-7, SWLS, Ecog-12, and DAS-17 Surveys.

| **Variables** | **GBP480** | | | **GBP240** | | | **% Difference** | **Absolute Delta** |
| --- | --- | --- | --- | --- | --- | --- | --- | --- |
|  | **Mean ± SD** | **% Change** (from baseline) | **Delta Change** (from baseline) | **Mean ± SD** | **% Change** (from baseline) | **Delta Change** (from baseline) | **GBP240 compared to GBP480** | **between groups** |
| **PSS-10**  **(Total Score)** | | | | | | | | |
| Baseline | 19.39±4.34 |  |  | 19.27±4.01 |  |  | -0.61% | -0.12 |
| Week 4 | 12.39±5.01 | -36.11% | -7.00* | 12.53±3.50 | -34.95% | -6.73* | 1.20% | 0.15 |
| **GAD-7**  **(Total Score)** | | | | | | | | |
| Baseline | 7.69±3.17 |  |  | 7.27±3.54 |  |  | -5.53% | -0.43 |
| Week 4 | 3.15±1.95 | -59.00% | -4.54* | 3.6±2.32 | -50.46% | -3.67* | 14.15% | 0.45 |
| **GAD-7**  **(Anxiety Resilience)** | | | | | | | | |
| Baseline | 0.69±0.48 |  |  | 1.07±0.26 |  |  | 54.08% | 0.37 |
| Week 4 | 0.23±0.59 | -66.67% | -0.46 | 0.6±0.63 | -43.75% | -0.4667 | 159.97% | 0.37 |
| **SWLS**  **(Total Score)** | | | | | | | | |
| Baseline | 18.62±7.57 |  |  | 20.53±6.64 |  |  | 10.30% | 1.91 |
| Week 4 | 25.77±6.60 | 38.43% | 7.15* | 23.73±6.96 | 15.58% | 3.20 | -7.90% | -2.03 |
| **Ecog-12**  **(Total Score)** | | | | | | | | |
| Baseline | 1.66±0.64 |  |  | 1.71±0.57 |  |  | 3.06% | 0.05 |
| Week 4 | 1.33±0.47 | -20.08% | -0.33 | 1.46±0.33 | -14.93% | -0.26 | 9.70% | 0.13 |
| **DAS-17**  *(***Total Score)** | | | | | | | | |
| Baseline | 45.08±14.67 |  |  | 51.87±25.65 |  |  | 15.06% | 6.79 |
| Week 4 | 42.39±15.78 | -5.97% | -2.69 | 43±20 | -17.10% | -8.87* | 1.45% | 0.62 |
| **DAS-17**  **(Perfectionism)** | | | | | | | | |
| Baseline | 26.31±9.41 |  |  | 31.53±17.67 |  |  | 19.86% | 5.23 |
| Week 4 | 24.15±10.16 | -8.19% | -2.15 | 25.067±12.77 | -20.51% | -6.47* | 3.78% | 0.91 |
| **DAS-17**  **(Dependency)** | | | | | | | | |
| Baseline | 18.77±6.83 |  |  | 20.33±8.99 |  |  | 8.33% | 1.56 |
| Week 4 | 18.23±7.26 | -2.87% | -0.5384 | 17.93±7.97 | -11.80% | -2.40 | -1.63% | -0.30 |

* p<0.05 within group from baseline, by paired samples t-test, Holm-Bonferroni correction for multiple comparisons; # p<0.05 between groups, by independent samples t-test, Holm-Bonferroni correction for multiple comparisons.

Abbreviations: GBP: Ginkgo biloba phosphatidylserine; 480: 480 mg daily; 240: 240 mg daily; PSS-10: Perceived Stress Scale; GAD-7: Generalize Anxiety Disorder; SWLS : Satisfaction with Life Scale; ECog-12: Everyday Cognition 12 Scale; DAS-17: Dysfunctional Attitudes Scale-17.

**Supplementary Table 2S.** Descriptive Statistics: SF-36.

| **Variables** | **GBP480** | | | **GBP240** | | | **% Difference** | **Absolute Delta** |
| --- | --- | --- | --- | --- | --- | --- | --- | --- |
|  | **Mean±SD** | **% Change (from baseline)** | **Delta Change (from baseline)** | **Mean±SD** | **% Change (from baseline)** | **Delta Change (from baseline)** | **GBP240 compared to GBP480** | **between groups** |
| **Physical Function** | | | | | | | | |
| Baseline | 84.23±17.03 |  |  | 86.33±16.95 |  |  | 2.50% | 2.10 |
| Week 4 | 87.38±16.45 | 3.74% | 3.15 | 91.66±12.05 | 6.18% | 5.33 | 4.90% | 4.28 |
| **Limits due to Physical Health** | | | | | | | | |
| Baseline | 63.46±45.20 |  |  | 83.33±32.27 |  |  | 31.31% | 19.87 |
| Week 4 | 75±39.52 | 18.18% | 11.53 | 95±19.36 | 14.00% | 11.66 | 26.67% | 20 |
| **Limits due to Emotional Health** | | | | | | | | |
| Baseline | 84.61±32.24 |  |  | 73.33±40.23 |  |  | -13.33% | -11.28 |
| Week 4 | 100±0 | 18.18% | 15.38 | 86.66±30.34 | 18.18% | 13.33 | -13.33% | -13.33 |
| **Energy/Fatigue** | | | | | | | | |
| Baseline | 43.84±17.33 |  |  | 46±18.24 |  |  | 4.91% | 2.15 |
| Week 4 | 71.53±12.31 | 63.16% | 27.69* | 64.33±16.67 | 39.86% | 18.33* | -10.07% | -7.20 |
| **Emotional Well-being** | | | | | | | | |
| Baseline | 61.23±16.92 |  |  | 68.26±13.97 |  |  | 11.49% | 7.03 |
| Week 4 | 83.07±8.50 | 35.68% | 21.84* | 80.26±12.78 | 17.58% | 12.00* | -3.38% | -2.81 |
| **Social Function** | | | | | | | | |
| Baseline | 73.07±17.56 |  |  | 75.8±21.43 |  |  | 3.73% | 2.72 |
| Week 4 | 91.34±11.84 | 25.00% | 18.26* | 89.16±11.44 | 17.63% | 13.36* | -2.39% | -2.17 |
| **Pain** | | | | | | | | |
| Baseline | 68.82±26.25 |  |  | 79.66±16.05 |  |  | 15.75% | 10.83 |
| Week 4 | 71.71±23.81 | 4.19% | 2.8846 | 84±16.08 | 5.44% | 4.3333 | 17.14% | 12.28 |
| **General Health** | | | | | | | | |
| Baseline | 61.15±18.61 |  |  | 63.33±21.26 |  |  | 3.56% | 2.17 |
| Week 4 | 68.07±19.84 | 11.32% | 6.92 | 72.66±15.45 | 14.74% | 9.33* | 6.74% | 4.58 |
|  | | | | | | | | |

* p<0.05 within group from baseline, by paired samples t-test, Holm-Bonferroni correction for multiple comparisons; # p<0.05 between groups, by independent samples t-test, Holm-Bonferroni correction for multiple comparisons.

Abbreviations: GBP: Ginkgo biloba phosphatidylserine; 480: 480 mg daily; 240: 240 mg daily; SF-36: Short Form 36.

**Supplementary Table 3S.** Descriptive Statistics: PERMA.

| **Variables** | **GBP480** | | | **GBP240** | | | **% Difference** | **Absolute Delta** |
| --- | --- | --- | --- | --- | --- | --- | --- | --- |
|  | **Mean ±SD** | **% Change (from baseline)** | **Delta Change (from baseline)** | **Mean ± SD** | **% Change (from baseline)** | **Delta Change (from baseline)** | **GBP240 compared to GBP480** | **between groups** |
| **Positive Emotion** | | | | | | | | |
| Baseline | 5.79±1.73 |  |  | 6.51±1.61 |  |  | 12.36% | 0.71 |
| Week 4 | 7.33±1.56 | 26.55% | 1.53* | 7.71±1.51 | 18.43% | 1.20* | 5.15% | 0.37 |
| **Engagement** | | | | | | | | |
| Baseline | 6.74±1.12 |  |  | 6.88±1.29 |  |  | 2.15% | 0.14 |
| Week 4 | 7.79±1.04 | 15.59% | 1.0513 | 7.57±0.97 | 10.00% | 0.68 | -2.79% | -0.21 |
| **Relationships** | | | | | | | | |
| Baseline | 6.21±2.32 |  |  | 7.68±1.28 |  |  | 23.78% | 1.47 |
| Week 4 | 7.71±1.47 | 24.25% | 1.50* | 8.28±1.33 | 7.80% | 0.60 | 7.40% | 0.57 |
| **Meaning** | | | | | | | | |
| Baseline | 6.67±1.48 |  |  | 7.27±1.52 |  |  | 9.27% | 0.61 |
| Week 4 | 8.02±1.35 | 20.50% | 1.36* | 7.91±1.07 | 8.70% | 0.63 | -1.43% | -0.11 |
| **Accomplishment** | | | | | | | | |
| Baseline | 6.30±1.64 |  |  | 6.88±1.57 |  |  | 9.21% | 0.58 |
| Week 4 | 7.48±1.98 | 18.70% | 1.17* | 7.62±1.33 | 10.64% | 0.73 | 1.80% | 0.13 |
| **PERMA Overall Wellbeing** | | | | | | | | |
| Baseline | 6.28±1.53 |  |  | 7.07±1.22 |  |  | 12.43% | 0.78 |
| Week 4 | 7.63±1.29 | 21.43% | 1.34* | 7.84±1.03 | 10.89% | 0.77* | 2.67% | 0.20 |
| **Negative Emotion** | | | | | | | | |
| Baseline | 4.17±1.84 |  |  | 4.31±2.07 |  |  | 3.15% | 0.13 |
| Week 4 | 3.25±2.22 | -22.09% | -0.923 | 3±1.628 | -30.41% | -1.31 | -7.87% | -0.25 |
| **Health** | | | | | | | | |
| Baseline | 6.33±2.23 |  |  | 6.97±1.47 |  |  | 10.17% | 0.64 |
| Week 4 | 7.05±2.19 | 11.33% | 0.71 | 7.57±1.26 | 8.60% | 0.60 | 7.47% | 0.52 |
| **Loneliness** | | | | | | | | |
| Baseline | 3.61±2.87 |  |  | 3.53±3.22 |  |  | -2.27% | -0.08 |
| Week 4 | 3.30±2.81 | -8.51% | -0.30 | 3.26±2.93 | -7.55% | -0.2666 | -1.24% | -0.04 |

* p<0.05 within group from baseline, by paired samples t-test, Holm-Bonferroni correction for multiple comparisons; # p<0.05 between groups, by independent samples t-test, Holm-Bonferroni correction for multiple comparisons.

Abbreviations: GBP: Ginkgo biloba phosphatidylserine; 480: 480 mg daily; 240: 240 mg daily; PERMA: Positive emotion, negative emotion, Engagement, Relationships, Meaning, and Accomplishment profile.

**Supplementary Table 4S.** Descriptive Statistics: POMS.

| **Variables** | **GBP480** | | | **GBP240** | | | **% Difference** | **Absolute Delta** |
| --- | --- | --- | --- | --- | --- | --- | --- | --- |
|  | **Mean±SD** | **% Change (from baseline)** | **Delta Change (from baseline)** | **Mean±SD** | **% Change (from baseline)** | **Delta Change (from baseline)** | **GBP240 compared to GBP480** | **between groups** |
| **Tension** | | | | | | | | |
| Baseline | 5.76±3.90 |  |  | 6.6±3.77 |  |  | 14.40% | 0.83 |
| Week 4 | 3.38±2.66 | -41.33% | -2.38 | 3.73±3.28 | -43.43% | -2.86* | 10.30% | 0.34 |
| **Anger** | | | | | | | | |
| Baseline | 5±5.43 |  |  | 3.2±3.76 |  |  | -36.00% | -1.80 |
| Week 4 | 2.15±3.67 | -56.92% | -2.84 | 2.4±2.99 | -25.00% | -0.80 | 11.43% | 0.24 |
| **Fatigue** | | | | | | | | |
| Baseline | 8.26±5.28 |  |  | 6.56±3.94 |  |  | -20.59% | -1.70 |
| Week 4 | 3.38±3.17 | -59.07% | -4.88* | 3.4±2.32 | -48.22% | -3.16* | 0.46% | 0.02 |
| **Depression** | | | | | | | | |
| Baseline | 4.61±4.67 |  |  | 4.26±3.76 |  |  | -7.56% | -0.34 |
| Week 4 | 2±3.53 | -56.67% | -2.61 | 1.73±1.98 | -59.38% | -2.53 | -13.34% | -0.26 |
| **Esteem-Related Affect** | | | | | | | | |
| Baseline | 13.23±4.22 |  |  | 15.2±4.34 |  |  | 14.88% | 1.96 |
| Week 4 | 16.76±3.89 | 26.74% | 3.53* | 17.2±3.70 | 13.16% | 2.00 | 2.57% | 0.43 |
| **Vigor** | | | | | | | | |
| Baseline | 5.69±3.66 |  |  | 7.6±4.51 |  |  | 33.51% | 1.901 |
| Week 4 | 9.30±3.22 | 63.51% | 3.61* | 9.93±4.19 | 30.70% | 2.33* | 6.72% | 0.62 |
| **Confusion** | | | | | | | | |
| Baseline | 4.57±3.14 |  |  | 4.86±3.06 |  |  | 6.33% | 0.28 |
| Week 4 | 2.23±2.24 | -51.26% | -2.34* | 2.86±1.99 | -41.10% | -2.00* | 28.51% | 0.63 |
| **Total Mood Disturbances. (TMD)** | | | | | | | | |
| Baseline | 111.15±24.80 |  |  | 103.66±21.35 |  |  | -6.74% | -7.48 |
| Week 4 | 88.07±19.37 | -20.76% | -23.08* | 87±15.24 | -16.08% | -16.67* | -1.22% | -1.07 |

* p<0.05 within group from baseline, paired samples t-test, Holm-Bonferroni correction for multiple comparisons; # p<0.05 between groups, by independent samples t-test, Holm-Bonferroni correction for multiple comparisons.

Abbreviations: GBP: Ginkgo biloba phosphatidylserine; 480: 480 mg daily; 240: 240 mg daily; POMS: Abbreviated Profile of Mood States; TMD: Total Mood Disturbance.
